# Factors affecting the simple febrile seizures in COVID-19 children: a case–control study from China

**DOI:** 10.3389/fneur.2023.1193843

**Published:** 2023-08-25

**Authors:** Haohao Wu, Kang Du, Xi Liang, Huijuan Fan, Ruiqiong Ba, Junsu Yang, Yue Wang

**Affiliations:** ^1^Department of Neurology, Qujing First People's Hospital, Yunnan, Qujing, China; ^2^Department of Paediatrics, Qujing First People's Hospital, Yunnan, Qujing, China

**Keywords:** COVID-19, simple febrile seizures, age, sex, hyponatremia

## Abstract

**Objective:**

The over-age phenomenon of simple febrile seizures (SFS) was found during the epidemic in COVID-19, but there was no clear explanation, especially in China. This study aimed to analyze the clinical and auxiliary examination features of SFS in children infected with the coronavirus disease 2019 (COVID-19).

**Methods:**

In total, 78 patients with SFS in the Department of Pediatric and Neurology of Qujing First People's Hospital were enrolled and divided into the COVID-19-positive group (case group) and the COVID-19-negative group (control group). The clinical characteristics, auxiliary examinations, and risk factors were analyzed.

**Results:**

There were significant differences in age stratification between the two groups. The proportion of children aged over 5 years old in the case group (47.4%) was higher than that of the control group (5%) (*p* < 0.0001). In terms of sex distribution, the proportion of males in the case group was higher than that in the control group (71.1% vs. 50%), but the difference was not statistically significant (*p* = 0.0678). For blood cell analysis, the values of white blood cells (WBC), lymphocytes (LY), and monocytes (MN) in the case group were significantly lower than those in the control group (*p* < 0.01). Serum electrolyte analysis showed the greatest difference in blood sodium. The proportion of hyponatremia in the case group was higher than that in the control group (36.8% vs. 17.5%), but the difference did not reach statistical significance (*p* = 0.0745). A multivariate logistic regression analysis showed that the history of FS was a independent protective factors for SFS in children with COVID-19 (OR = 0.115, *p* = 0.009), and age was an independent risk factor for SFS in children with COVID-19 (OR = 1.042, *p* = 0.001).

**Conclusion:**

Age distribution, sex a previous history of FS and hyponatremia were different between children with and without COVID-19 in SFS. The history of FS was an independent protective factors for SFS in children with COVID-19.

## Introduction

Simple febrile seizures (SFS) is defined as a short-time (<15 min) generalized seizure caused by non-nervous system acute diseases in a febrile course (anal temperature ≥38.5°C and axillary temperature ≥38°C), with no recurrence or neurological dysfunction (such as Todd's palsy) within 24 h as well as no history of febrile convulsion (1).

SFS is previously thought to occur mostly in children between the ages of 6 months and 5 years (1), but since the coronavirus disease 2019 (COVID-19) pandemic, there have been multiple cases of FS in children in the high age group (>5 years). According to the guidelines, children over 5 years of age with FS are more prone to be diagnosed as complex FS (2), which is characterized by a long attack time or a focal attack and recurs within 24 h. After the attack, there may be abnormal manifestations of the nervous system, such as Todd's paralysis. However, these children lack the evidence of nervous system diseases, neurological dysfunction, long-term onset (≥15 min), and abnormal electroencephalogram, so they do not conform to the diagnosis of complex FS but SFS. Studies by scholars from multiple countries have found (3–5) that FS of multi-ethnic COVID-19-infected children in many countries, including South Africa, Japan, South Korea, and Europe, occurs beyond the conventional age range, that is, children younger than 6 months and older than 5 years old. They have the typical clinical characteristics of SFS, but not the typical age range, so they may have a “late-onset SFS.” However, this significant age difference has not been fully explained by a mechanism or analyzed by the risk factors, and studies targeting the population in China have not been reported to date.

In this study, the general information, clinical features, and auxiliary examinations of SFS children infected with COVID-19 were collected and compared with those SFS children not infected with COVID-19 in order to find the association between COVID-19 and SFS in children in China, identify its risk factors, and improve the understanding of clinicians on the characteristics of SFS in children with COVID-19.

## Materials and methods

### Subjects and clinical data

A total of 78 cases of children with SFS in China in the inpatient Department of Pediatrics and Neurology of Qujing First People's Hospital were collected from June to December 2022. All the children met the inclusion criteria of this study: All children met the diagnostic criteria of SFS, but because the focus of this study was on the elederly children of SFS, age was not regarded as the absolute inclusion criteria, and all the children were randomly enrolled in the study and did not match any of the items in the exclusion criteria. The sex, age, axillary temperature, duration of convulsion, onset time, previous history of FS, family history, blood cell analysis (white blood cells, neutrophils, lymphocytes, and monocyte values), serum electrolytes (potassium, sodium, chloride, and calcium), etiology detection, lung CT, and cranial MRI were recorded. According to the results of COVID-19 nucleic acid detection, they were divided into a COVID-19-positive group (case group) and a COVID-19-negative group (control group). They were divided into the younger age group (≤ 5 years) and senior age group (>5 years) according to age.

### Inclusion and exclusion criteria

Inclusion criteria were set following the Diagnosis and Management of Febrile Seizures Expert Consensus (2016) (2) with Children with SFS: axillary temperature ≥38°C; generalized seizure; duration <15 min; one febrile course and one attack during 24 h; absence of acute systemic metabolic abnormality that may provoke convulsions. There were no abnormal neurological signs. Special note: Age of onset (6 months to 5 years) was not considered an absolute inclusion criterion in this study.

The exclusion criteria were as follows: focal seizure; with epilepsy or a history of febrile convulsions; status epilepticus convulsive; combined central nervous system diseases or signs; electroencephalogram showing obvious background abnormality or epileptic discharge; patients with a severe mental disorder or consciousness disorder who cannot cooperate with the examination; other diseases or drugs that can lead to seizures; and families or patients who declined to participate in the study.

### Detection of COVID-19

The nucleic acid of COVID-19 was detected using a 2019 novel coronavirus (ORF1ab/N gene) nucleic acid detection kit (double fluorescent PCR), and pharyngeal swabs were collected. Other blood and etiology detection and auxiliary examination were carried out by the related departments of the hospital.

### Statistical analysis

Continuous variables were presented as median (Q1, Q3). The continuous variables showed an abnormal distribution (as evaluated using the single-sample K-S test), and the Mann–Whitney U-test was used for evaluating differences in continuous variables between the two groups. The categorical data were expressed as the number of cases or the constituent ratio (%), and the chi-square test or Fisher's exact test was applied. The “positive COVID-19” were dichotomized into two dependent variables (presence/absence of positive COVID-19 = 1/0). Risk factors of positive COVID-19 were analyzed by multivariable logistic regression, and independent variables were selected using the stepwise method. Two-sided *p*-values were calculated for all analyses; a *p*-value of < 0.05 was considered to be statistically significant. Statistical analysis was performed with SPSS Statistics version 24 (IBM, Armonk, NY, USA) and GraphPad Prism version 8.00 (GraphPad Software, San Diego, CA, USA).

## Results

### Clinical characteristics

A total of 78 cases were included in the study, including 38 cases in the case group, 20 cases in the younger age group, and 18 cases in the advanced age group, with 27 males and 11 females. There were 40 cases in the control group, including 38 cases in the younger age group, and 2 cases in the advanced age group, with 20 males and 20 females. The proportion of the younger age group in the case group (52.6%) was lower than that of the control group (95%), and the difference was statistically significant (*p* < 0.0001). The proportion of the case group in the aged group (47.4%) was higher than that of the control group (5%) (*p* < 0.0001). The proportion of males in the case group (71.1%) was significantly higher than that in the control group (50%), but the difference was not statistically significant (*p* = 0.0678) (Table 1).

**Table 1:** Comparison of demographic characteristics, clinical features and ancillary examinations between two groups.

**Characteristics**	**Total (*n =* 78)**	**COVID-19 positive (*n =* 38)**	**COVID-19 negative (*n =* 40)**	***P* value**
Age at presentation, m				
>5y *n* (%)	20 (25.6)	18 (47.4)	2 (5.0)	**< 0.0001** ^***^
≤ 5y *n* (%)	58 (74.4)	20 (52.6)	38 (95.0)	**< 0.0001** ^***^
Male, *n* (%)	47 (60.2)	27 (71.1)	20 (50.0)	0.0678
History of FS, *n* (%)	20 (25.6)	6 (15.8)	14 (35.0)	0.0704
Fever peak, °C	39.0 (38.6, 39.5)	39.1 (38.6, 39.6)	39.0 (38.6, 39.5)	0.5147
Duration of seizures^#^, minutes	3.0 (1.0, 4.5)	3.0 (1.0, 4.5)	2.5 (0.6, 4.8)	0.4313
Number of seizures, median (range)	1.0 (1.0, 5.0)	1.0 (1.0, 5.0)	1.0 (1.0, 3.0)	0.3245
Laboratory results				
WBC (× 10^9^/L)	6.3 (4.6, 8.3)	4.8 (3.6, 6.4)	7.8 (5.5, 10.3)	**< 0.0001** ^***^
NE (× 10^9^/L)	3.1 (2.0, 5.5)	2.6 (1.7, 4.8)	3.7 (2.3, 6.5)	0.179
LN (× 10^9^/L)	1.7 (1.1, 2.7)	1.4 (1.0, 1.8)	2.6 (1.4, 4.4)	**< 0.0001** ^***^
MN (× 10^9^/L)	0.5 (0.3, 0.7)	0.4 (0.3, 0.5)	0.6 (0.4, 0.8)	**0.0022** ^**^
PCT (ng/mL)	0.1 (0.1, 0.4)	0.2 (0.1, 0.3)	0.1 (0.1, 0.5)	0.3241
CRP (mg/L)	9.6 (4.1, 24.9)	9.2 (3.9, 19.8)	9.9 (4.3, 27.7)	0.451
Serum kalium decreased, *n* (%)	2 (2.6)	2 (5.3)	0 (0)	0.2341
Serum sodium decreased, *n* (%)	21 (26.9)	14 (36.8)	7 (17.5)	0.0745
Serum chlorine decreased, *n* (%)	2 (2.6)	2 (5.3)	0 (0)	0.2341
Serum calcium decreased, *n* (%)	0 (0)	0 (0)	0 (0)	>0.9999
Etiology detection				
Streptococcal infection, *n* (%)	2 (2.5)	2 (5.3)	0 (0)	0.2341
Mycoplasma pneumoniae infection, *n* (%)	8 (10.2)	2 (5.3)	6(15.0)	0.264
Negative, *n* (%)	65 (83.3)	34 (89.4)	31 (77.5)	0.2259
Lung CT				
Bronchopneumonia or mild pneumonia, *n* (%)	22 (28.3)	13 (34.3)	9 (22.5)	0.3169
Head MRI				
Myelin sheath immature, *n* (%)	5 (6.5)	0 (0)	5 (12.5)	0.0549

CRP, C-reactive protein; FS, febrile seizures; LN, lymphocytes; MN, monocytes; NE, neutrophil; PCT, procalcitonin; WBC, white blood cells. Continuous variables were presented as median (interquartile range) except for being specially marked; ^#^The data of only 53 cases were available, including 21 cases in COVID-19 positive group and 32 cases in COVID-19 negative group; ^**^, ^***^Significant correlation at 0.01 and 0.001 level, respectively. All significant P values are printed in bold.

There was no statistical difference in axillary temperature, seizure duration, seizure frequency, or family history between the two groups (*p* > 0.05). The proportion of patients with previous FC history in the case group (15.8%) was lower than that in the control group (35%), but the difference did not reach statistical significance (*p* = 0.0704) (Table 1).

### Analysis of auxiliary examinations

Just as Table 1 showed, the median white blood cells (WBC), neutrophils (NE), lymphocytes (LN), and monocytes (MN) were 4.8 × 10^9^/L, 2.6 × 10^9^/L, 1.4 × 10^9^/L, and 0.4 × 10^9^/L in the case group, respectively, which were significantly lower than that in the control group with WBC 7.8 × 10^9^/L (*p* < 0.0001), LN 2.6 × 10^9^/L (*p* < 0.0001), MN 0.6 × 10^9^/L (*p* = 0.0022), except for NE with 3.7 × 10^9^/L (*p* = 0.179). There was no statistical difference in serum procalcitonin (PCT) and C-reactive protein (CRP) levels (*p* > 0.05).

**Figure 1:**
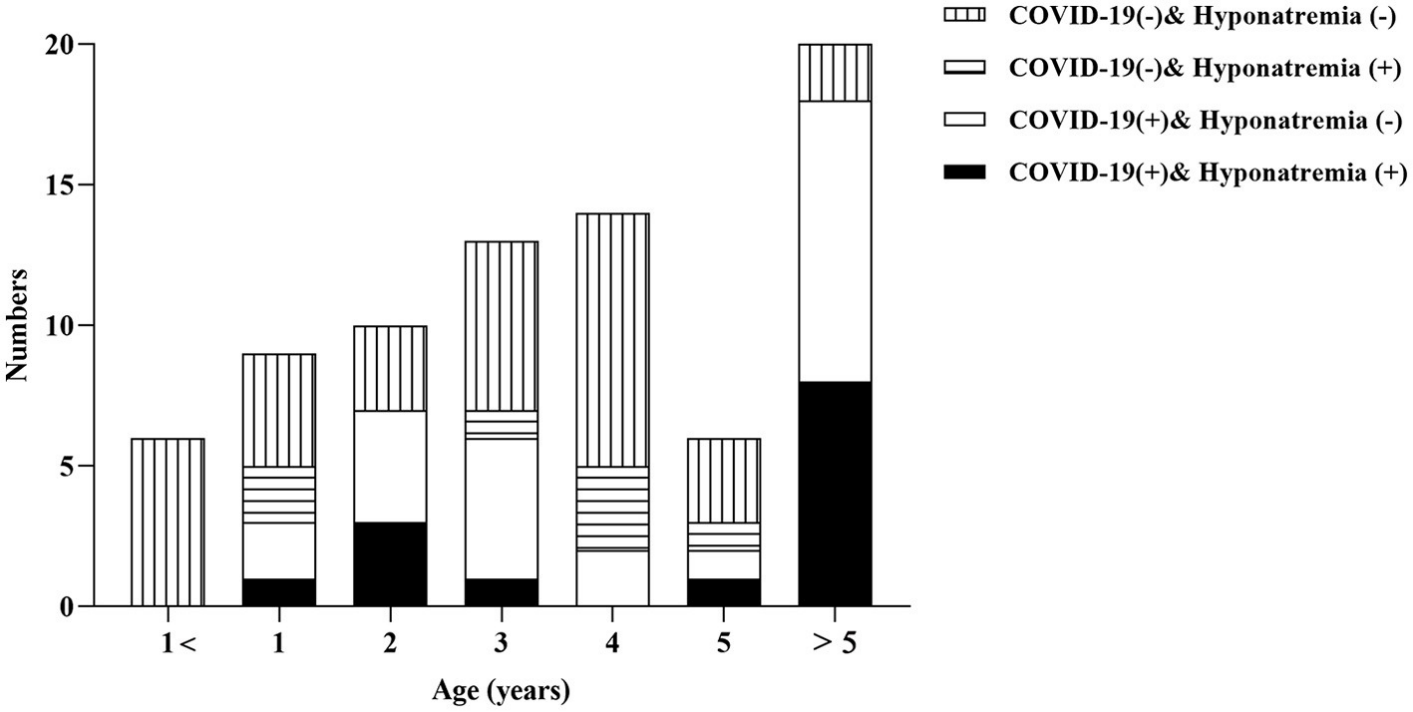
Histogram of age distribution with COVID-19 and hyponatremia.

Analysis of the electrolyte test showed that the incidence of hyponatremia was more common than other electrolyte disorders in the case group, which was higher than that in the control group (36.8 vs. 17.5%). However, a significant difference was not observed (*p* = 0.0745) (Table 1). Furthermore, it was found that hyponatremia combined with COVID-19 was the most common in the age >5 years old group (*n* = 8, 57.1%), while hyponatremia in non-COVID-19 children was relatively rare in all age groups (all *n* = 7), and most of them occurred in children aged approximately 4 years old (*n* = 3) (Figure 1).

Etiological analysis showed that two cases of combined streptococcus infection and two cases of mycoplasma pneumoniae infection were found in children with COVID-19, but there was no statistical difference as compared with that in the control group (*p* > 0.05) (Table 1).

Lung CT revealed that lung lesions were more common (34.3%) in the case group, mainly including pulmonary ground-glass nodules, inflammatory streak lesions, enhanced lung texture, and signs of bronchopneumonia and exudation. Compared with the control group (22.5%), the symptoms were more severe, but the difference was not statistically significant (*p* > 0.05). No abnormality was observed in the case group on brain MRI, but five cases with immature myelin sheath development were found in the control group with no statistically significant difference between the two groups (*p* = 0.0549) (Table 1).

### Analysis of risk factors

The multivariate logistic regression analysis (Table 2) showed that age was an independent risk factor for SFS in children with COVID-19 (OR = 1.042, *p* = 0.001), while previous FS history negatively correlated with the COVID-19-positive group and was a risk factor for SFS in children without COVID-19 (OR = 0.115, *p* = 0.009).

**Table 2:** Multivariable logistic regression model for variables associated with COVID-19 positive in SFS children.

**Variables**	**β**	**OR**	**95%Cl**	***P*-value**	
Sex	0.975	2.651	0.825	8.515	0.102
Age	0.041	1.042	1.018	1.067	**0.001** ^***^
Hyponatremia	1.086	2.963	0.828	10.603	0.095
History of FS	−2.164	0.115	0.023	0.58	**0.009** ^**^

FS, febrile seizures. ^**^, ^***^Significant correlation at 0.01 and 0.001 level, respectively. All significant *P* values are printed in bold.

## Discussion

FS is a common paroxysmal and self-limited childhood disease in pediatrics and neurology department and has a significant relationship with fever (anal temperature ≥38.5°C and axillary temperature ≥38°C), among which SFS is the most common (70–80%), and SFS is a highly self-limited and age-dependent FS. The age of onset is 6 months to 5 years old, and the prognosis is favorable. On the contrary, complex FS mostly occurs at <6 months old or >5 years old (2). As for the age of onset of SFS, there has always been controversy. A Japanese study found (6) that very few cases of SFS occur in late childhood (>5 years old) and named it “late FS”. Therefore, SFS and complex FS cannot be distinguished by age distribution alone.

During the epidemic period of megalomania, COVID-19-infected individuals presented with a series of nervous system symptoms, the main mechanism of which is that COVID-19 invades host cells (7) by binding to angiotensin-converting enzyme 2 and can be expressed on the surface of various nerve cells (8), causing headache, dizziness, olfactory dysfunction, cognitive impairment, convulsion, status epilepticus, paralysis, and other symptoms (9). Of interest, COVID-19-induced FS in children seems to lack typical age dependence. A Swedish study (9) found that among the three COVID-19 omicron variant children, one was only 3 months old, and the child had no neurological signs previously or in the family history. CRP, cranial imaging, cerebrospinal fluid, and electroencephalogram were all normal, so he did not conform to the characteristics of complex FS but showed SFS. Another child with COVID-19 omicron was 14 years old, and his medical history was basically the same as that of the 3-month-old child. However, this child had no fever and only symptoms of upper respiratory tract infection. Although it did not meet the diagnosis of FS, this child also had seizures that could not be explained by other causes. Another South Korean study has found (4) that patients with FS with COVID-19 were often male-dominated, and they were older than those who were not infected with COVID-19. However, studies on the China population have not been reported so far.

This study found that the onset age of children with COVID-19 associated with SFS in China also presented a significant late-onset phenomenon similar to studies in other countries. For the case group, a total of 18 cases were in the >5-year-old age group, accounting for 47.4%, and 6 of them experienced FS previously; that is, 12 cases were the first-episode SFS in the elederly group, which was significantly different from non-COVID-19 children with SFS. However, no literature clarified the pathogenesis of SFS in older children caused by COVID-19, and no evidence showed that these patients had significant brain lesions. Therefore, we speculated that it might be the indirect neuroimmune response of the brain caused by the inflammatory storm of COVID-19, which induced the onset of the disease in these older children who should not have SFS. The pathological mechanism needed further studies. At the same time, the sex composition of COVID-19 with SFS was dominated by male patients, with the ratio of male to female being 2.45: 1 instead of the equal ratio of male to female of non-COVID-19 with SFS (1: 1). However, the difference between the two groups was not statistically significant, which was considered to be related to the insufficient sample size.

Some Japanese scholars compared the FS data before and after the COVID-19 pandemic and found that most of the children infected with COVID-19 were non-critical patients with FS, whose manifestations were comparable to the FS symptoms of non-COVID-19 (5). Based on the data reported by European scholars from two atypical-age children with FS infected with COVID-19 (9), it was found that the clinical symptoms of the two children were not different from the typical clinical manifestations of SFS.

This study found that the SFS of children with COVID-19 was comparable to that of non-COVID-19 in axillary temperature, seizure duration, seizure frequency, and family history, while the proportion of previous FS history (15.8%) was lower than that of non-COVID-19 children (35%), and the difference was not statistically significant, similar to the studies reported above (5, 9). However, it was worth noting that the previous FS history was an independent risk factor for SFS in children with non-COVID-19 in our study, indirectly suggesting that even in children with COVID-19 infection without previous history of FS, COVID-19 might also induce SFS. In fact, the vast majority of children with SFS caused by COVID-19 were in good health in the past. In this study, only 15.8% of children with a previous history of FS were observed, and the cranial MRI was normal, inferring SFS in children with COVID-19 might not be related to previous history of nervous system diseases and cranial MRI abnormalities. This phenomenon was similar to acute necrotizing encephalopathy (ANE) in children with COVID-19 (10, 11). Many studies have confirmed that the vast majority of cases of COVID-19-related ANE in children have been in good health before onset.

During the initial infection, COVID-19 mainly attacked the human immune system, resulting in “inflammatory storm,” in which new virus particles synthesized under the action of ACE2 were released to the outside of the cells, damaging the lung capillaries, and then resulting in the reduction of lung connexins and the enlargement of alveolar space. The increase of blood flow from the capillaries led to the decline of leukocytes and increased apoptosis of lymphocytes. Therefore, in the early stage of COVID-19 infection, the blood cell analysis of patients mainly showed the decline of leukocytes and lymphocytes, which was dominated by the decline of lymphocyte value (12, 13). This result was basically consistent with the result of this study. In this study, the blood cells of children with COVID-19 were analyzed, and the decreased values of white cells and lymphocytes were significantly different from those of children with non-COVID-19 infection.

However, whether these differences in inflammatory cells contribute to the susceptibility to SFS is still unclear. A cerebrospinal fluid examination was performed in some patients, and the results of all of them were normal, so there might be an indirect effect of COVID-19 on inflammation of the nervous system. Studies have shown that COVID-19 had an indirect effect on the central nervous system, such as an indirect inflammatory immune response (14). They found that COVID-19 was not detected in the brain tissue of patients, but monocyte infiltration and extensive glial cell activation led to the upregulation of inflammatory factors in the nervous system and inflammatory damage in postmortem brains from nine ill COVID-19 patients. Although it could be explained that COVID-19 has an indirect effect on central nervous system inflammation, there is still a lack of direct evidence for its induction of FS. Therefore, more research studies are still needed to confirm the correlation between inflammatory cells and SFS in children with COVID-19.

Hyponatremia is closely related to epileptic seizure and some studies (15) have shown that with the decline of sodium level, the risk of epileptic seizure increases gradually. The probability of hyponatremia in COVID-19 is higher, and it may be a risk factor for poor prognosis of patients with COVID-19 (16). Therefore, whether COVID-19-associated hyponatremia causes the susceptibility of children to SFS is of great concern. In this study, we found that the incidence of hyponatremia in children infected with COVID-19 (36.8%) was significantly higher than that in children not infected with COVID-19 (17.5%), but the difference was not statistically significant (*p* = 0.0745). Similarly, there was no statistical difference in the multivariate logistic regression analysis, which was considered to be related to the insufficient sample size, and the cases could be further expanded in future studies. In addition, we found that the combination of COVID-19 and hyponatremia was most common in the age group >5 years old. However, the mechanism of SFS in older children caused by COVID-19 still needs to be further verified.

SFS is mainly related to fever caused by a variety of infectious diseases, especially viral infection. However, the true pathogenesis of SFS is still unclear, which may be related to immature myelin sheath development and genetic susceptibility (17, 18). In this study, we found that five cases (12.5%) of non-COVID-19 children with SFS developed myelin immaturity, but no abnormal manifestation of cranial MRI was seen in COVID-19 children, indicating that it did not mean that SFS would not occur in COVID-19 children with normal cranial imaging findings. This may be a difference in the etiology of SFS caused by COVID-19 alone.

In summary, SFS of children with COVID-19 was obviously “over-aged,” and age was an independent risk factor for SFS in children with COVID-19. At the same time, the proportion of male children infected with COVID-19 was higher, and the decline in white blood cells and lymphocytes was more common. The majority of SFS in children with COVID-19 were found in patients who were healthy in the past, with normal head MRIs. On the contrary, the previous history of SFS was mostly found in children with non-COVID-19 and was an independent risk factor, and several cases of myelin dysplasia were also found in the head MRIs of these children with non-COVID-19. It suggested that we should be highly alert to the possibility of COVID-19 infection when children have SFS in the senior age group, especially male children. In addition, combining blood cell analysis, past history, and head imaging information could provide more powerful evidence to help clinicians judge the possibility of COVID-19 infection in children at an early stage and investigate it in time. The incidence of hyponatremia in children with COVID-19 was high, but whether to participate in the mechanism of SFS needs further study in the later stage.

The study also has some limitations. First, the study included a small number of cases, which was mainly due to the local epidemic situation and the detection rate of COVID-19 nucleic acid in children. In addition, this study mainly aimed at the clinical manifestations, routine tests, and examinations of children with COVID-19 complicated with SFS and had not carried out a detailed research on the pathogenesis. These limitations can be further improved in future research.

## Data availability statement

The original contributions presented in the study are included in the article/supplementary material, further inquiries can be directed to the corresponding author.

## Ethics statement

The studies involving humans were approved by the Ethic Committee of Qujing First People's Hospital. The studies were conducted in accordance with the local legislation and institutional requirements. Written informed consent for participation in this study was provided by the participants' legal guardians/next of kin.

## Author contributions

HW: acquisition of data, completion of statistical analysis, drafting of the initial manuscript, and writing of the final manuscript. KD: acquisition of data, study concept and design, completion of statistical analysis, and critical revision of the manuscript. XL, HF, RB, and JY: study concept and design and critical revision of the manuscript. YW: data review, interpretation of results, and revision of the initial draft. All authors contributed to the article and approved the submitted version.
